# Subjective Age as a Moderator in the Reciprocal Effects Between Posttraumatic Stress Disorder Symptoms and Self-Rated Physical Functioning

**DOI:** 10.3389/fpsyg.2018.01746

**Published:** 2018-09-13

**Authors:** Amit Shrira, Yuval Palgi, Yaakov Hoffman, Sharon Avidor, Ehud Bodner, Menachem Ben-Ezra, Moshe Bensimon

**Affiliations:** ^1^The Interdisciplinary Department of Social Sciences, Bar-Ilan University, Ramat-Gan, Israel; ^2^Department of Gerontology, The Center for Research and Study of Aging, University of Haifa, Haifa, Israel; ^3^Department of Social and Community Sciences, Ruppin Academic College, Emek Hefer, Israel; ^4^Department of Music, Bar-Ilan University, Ramat-Gan, Israel; ^5^School of Social Work, Ariel University, Ariel, Israel; ^6^Department of Criminology, Bar-Ilan University, Ramat-Gan, Israel

**Keywords:** PTSD, self-rated physical functioning, subjective age, recovery capital, older adults, missile attacks

## Abstract

It is now widely acknowledged that physical decline may increase among middle-aged and older adults who suffer from posttraumatic stress disorder (PTSD). Much less is known about the temporal sequencing of PTSD and physical decline relationship over time. While PTSD can lead to physical decline, physical decline may preserve or augment existing PTSD symptoms. Both problems can also mutually affect each other forming a vicious cycle. Additionally, it is important to address variables that can mitigate these longitudinal effects. Following the recovery capital framework, we consider how the existence or lack of capital in the form of young age identity may affect the recovery process. Therefore, the current study aimed to examine the reciprocal effects of PTSD symptoms and self-rated physical functioning and further test whether one’s subjective age moderates these effects. Using in-region random digit dialing, we collected a stratified sample of community dwelling older adult residing in south Israel. Of that sample (*N* at T1 = 339), 132 older adults (age range = 51–88, mean age = 66.90, *SD* = 9.14) were interviewed 4 months after the 2014 Israel–Gaza conflict (T2) and 1 year later (T3). Participants responded to PTSD symptoms scale, and reported their physical functioning and subjective age. PTSD symptoms and self-rated physical functioning were tested as both predictors and outcomes in a cross-lagged model. The moderating effect of subjective age was assessed by examining whether T2 variables interacted with subjective age in predicting T3 outcomes. Results showed that higher PTSD symptoms at T2 were associated with subsequent lower self-rated physical functioning at T3, yet self-rated physical functioning at T2 did not predict PTSD symptoms at T3, thereby highlighting the PTSD self-rated physical function direction. Moreover, subjective age moderated this latter association, so that this relationship was significant only for those who felt relatively older. In addition to clarifying the temporal sequencing of the PTSD self-rated physical functioning association, the study further suggests that an older subjective age (i.e., lack of recovery capital) could render middle-aged and older adults more susceptible to physical decline following PTSD symptoms. We therefore propose to develop interventions aimed at coping with an older age identity and facilitating a younger age identity among traumatized older individuals.

## Introduction

According to recent models, posttraumatic stress disorder (PTSD) is the primary pathway through which traumatic exposure leads to physical health impairments and accelerated aging (Schnurr, 2017). Physical health impairment has been observed among posttraumatic individuals across a continuum of measures that range from self-reported physical symptoms to physician-diagnosed health-care disorders, biological markers, and mortality (Pacella et al., 2013; Solomon et al., 2014; Lohr et al., 2015; Williamson et al., 2015; Cook and Simiola, 2017). Yet the direction of these effects (i.e., PTSD physical impairment or *vice versa*) remains ambiguous. Thus, this study focuses on the temporal sequencing of the well-documented association between PTSD and adverse physical health. The study further assesses potential moderators of this temporal sequencing by focusing on the role of subjective age.

Posttraumatic stress disorder is thought to be connected to physical health outcomes *via* psychological, biological, behavioral, and attentional mechanisms (Schnurr, 2017). Psychological mechanisms that are catalysts to poor physical health include the features of PTSD itself, such as re-experiencing the traumatic event, avoidance of stimuli and feelings reminiscent of the trauma, increased arousal, and negative alterations in cognitions and mood (American Psychiatric Association, 2013), as well as additional broader psychological effects, such as avoidant strategies, hostility, and depression (Schnurr and Green, 2004). Biological mechanisms include dysregulations in the locus coeruleus-noradrenergic and the hypothalamic–pituitary–adrenal systems following PTSD, linking this major psychopathology to numerous bodily dysfunctions, such as high blood pressure, decreased heart rate variability, and dysregulated metabolism of insulin, glucose, and lipids (Yehuda et al., 2015). Behavioral mechanisms comprise risk behaviors concomitant to PTSD that increase one’s susceptibility to physical disease, such as smoking and intake of alcohol and drugs (Rheingold et al., 2004). PTSD further reduces preventive behaviors such as physical activity, diet, and regular utilization of medical care, possibly due to dampened motivation for proactive measures and low self-efficacy (Lee and Park, 2018). Finally, somatization or alternatively decreased attention to medical problems due to dissociation are amongst some of the attentional mechanisms linking between PTSD and decreased physical health (Schnurr and Green, 2004).

Although PTSD can catalyze physical dysfunction, the opposite direction is also possible, i.e., physical morbidity kindles PTSD symptoms or exacerbates existing symptoms. For example, a study in war injured veterans showed a robust association between level of injury and PTSD symptom severity that subsequently emerged (Grieger et al., 2006). A reciprocal relationship between these factors is also possible, namely, that both problems mutually affect each other forming a vicious cycle. The reciprocal effects of PTSD and physical health are especially pertinent in old age when physical decline is common. Nevertheless, the potential reciprocal effects between PTSD symptoms and physical health were rarely examined, and when they were, it was mainly in samples of young adults (e.g., Ramchand et al., 2008; Schweininger et al., 2015; Valentine et al., 2016).

The few studies that did examine reciprocal effects mostly pointed to the possibility that PTSD symptoms lead to physical conditions, whereas physical impairment is less likely to precede PTSD. In one study extending over a 22-year follow-up period, PTSD symptoms predicted type 2 diabetes among adult and older adult women, but the reverse effect leading from diabetes to PTSD was not supported (Roberts et al., 2015). Similarly, in an assessment of prospective effects of PTSD symptoms and physical disability among young adult hospital patients admitted after injury, Schweininger et al. (2015) found that disability after the injury did not predict 12-month psychopathology, but PTSD symptoms did predict subsequent physical disability. In yet another study on young adults who survived motor vehicle collisions, PTSD symptoms at 4 weeks post-accident predicted higher bodily pain 3 months later, yet physical health symptoms at 4 weeks post-accident were not predictive of later PTSD (Valentine et al., 2016). Finally, in a sample of young adults exposed to community violence, physical functioning prior to traumatic exposure was not related to subsequent PTSD symptoms, whereas PTSD symptoms predicted physical functioning 3 months later (Ramchand et al., 2008). Nevertheless, in a longer follow-up of up to 1 year, the reverse patterns emerged, whereby physical functioning predicted PTSD symptoms. Therefore, the authors conclude that PTSD and physical functioning have reciprocal effects.

In addition, none of the previous studies accounted for the possibility that the reciprocal effects might be mitigated or intensified by additional variables. In this paper, we focus on the moderating role of subjective age, examining its effects on the reciprocal relationships between PTSD and self-rated physical functioning.

Subjective age refers to how old one perceives oneself to be (e.g., Kotter-Grühn et al., 2015; Stephan et al., 2015a). Diehl et al. (2014) suggest that subjective age is included in an overarching self-related awareness focused on age and aging resulting in a form of self-representation – attributes that are part of a person’s self-understanding and self-knowledge. In later adulthood, this self-representation becomes an integral part of a person’s overall self-concept and identity. Subjective age and self-esteem are correlated, yet hold a relatively small amount of shared variance (Westerhof et al., 2012), suggesting that the former represents a specific part of one’s self-concept. Subjective age is mostly assessed using a single item (e.g., “How old do you feel?”) which is strongly associated with questionnaires that capture different dimensions of subjective age (e.g., felt age, look age, act age; Barak, 1987; Cleveland et al., 1997). Subjective age is most likely a unidimensional construct, as the different domains of subjective age tend to converge together and load on a single factor (Barak, 2009).

A young subjective age, or feeling younger than one’s chronological age, is considered an adaptive perception in a society that often devalues old age. Accordingly, older adults indeed generally feel younger than their current age, and the tendency to feel younger than one’s age increases across the lifespan (Chopik et al., 2018). Feeling relatively older on the other hand could indicate greater perceived vulnerability to age-related decline in health (Kotter-Grühn and Hess, 2012). Relatedly, the stereotype embodiment theory (Levy, 2009) maintains that aging adults gradually internalize the stereotypical views society holds against older adults, as well as their own personal negative views. When these views become more relevant as the individual ages, they begin to operate unconsciously through multiple pathways, and negatively affect the individual’s health. An older age identity can be seen as an internalization of negative (often health-related) age stereotypes.

In the specific context of traumatic exposure and PTSD, subjective age can be conceptualized within the recovery capital framework (Granfield and Cloud, 1999, 2001). Recovery capital denotes the internal and external resources that enable individuals to initiate and sustain recovery. Accordingly, “capital” should be understood as a body of resources that can be accumulated or exhausted. Later, Cloud and Granfield (2008) revisited their initial concept and have argued that there are four components to recovery capital: cultural capital, or the values and beliefs associated with cultural group membership; physical capital, including financial assets and status, especially housing and shelter, clothing, and food; human capital, which comprises the acquired and inherited traits, such as knowledge, skills, and mental health as well as personal values and beliefs; and social capital, which includes close, especially family, relationships that are supportive of recovery efforts. These four components of recovery capital provide a comprehensive framework for understanding the wide range of resources that can benefit the effort to overcome and recover from various kinds of adversities.

A young subjective age may be considered as recovery capital reflecting a perception that one has greater resources than the demands brought on by traumatic events (Hoffman et al., 2016; see also Stephan et al., 2011). Following, an old age identity may reflect a perception that one is not equipped to handle traumatic exposure and its aftermath. Accordingly, higher PTSD symptom levels were found among those who hold an older age identity (Solomon et al., 2009; Avidor et al., 2016; Hoffman et al., 2016; Palgi, 2016).

In addition, perceiving oneself as older than one’s age could moderate the reciprocal effects between PTSD symptoms and physical functioning, and amplify these effects *via* several psychological, biological, behavioral, and attentional mechanisms. First, an older subjective age is related to symptoms of depression and anxiety (Shrira et al., 2014), and therefore can further burden the diminished psychological resources of posttraumatic older adults. Second, feeling older is related to various biological markers of inflammation (Stephan et al., 2015b), kidney failure (Stephan et al., 2017), and accelerated senescence (Lahav et al., 2018), and thereby have the potential to aggravate the noxious effects of PTSD on bodily systems. Third, one’s self-perception of old age may further inhibit help-seeking behaviors, harm health-preserving behaviors, and worsen health-risk behaviors in addition to already existing risk-behaviors due to PTSD (Wienert et al., 2017). Finally, both PTSD symptoms and low physical functioning could be viewed more negatively and bring about more distress due to internalized age stereotypes reflected by an older age identity (Kotter-Grühn and Hess, 2012).

Several studies demonstrated that subjective age can serve as a moderating variable that impacts associations between, for example, PTSD/distress and other variables. Thus, an older age identity strengthened the relationship between PTSD symptoms and successful aging (Shrira et al., 2016), and was related to higher levels of distress as predicted by subjective nearness-to-death (Shrira et al., 2014). Nevertheless, these studies were cross-sectional and therefore could not assess reciprocal effects. The only study known to us that examined subjective age as a moderator of reciprocal effects showed that the effect of depressive symptoms on physical morbidity a decade later was stronger among adults and older adults who felt older at baseline. Yet, subjective age did not moderate the reverse effect, meaning that of baseline physical morbidity on subsequent depressive symptoms (Segel-Karpas et al., 2017).

Following the above literature, we first hypothesize that PTSD symptoms and self-rated physical functioning will have reciprocal effects. Thus, higher PTSD symptoms will predict lower self-rated physical functioning, and higher self-rated physical functioning will predict lower PTSD symptoms over time. Although the latter effect has received more support in the literature than the former, there are still some indications that reciprocal effects may exist. Moreover, we further hypothesize that the reciprocal effects between PTSD symptoms and self-rated physical functioning will be stronger among those feeling older.

## Materials and Methods

### Participants and Procedure

Using a polling company, we sampled, through an in-region random digit dialing methodology, Jewish participants aged 50 or above from the national telephone directory, residing in the south of Israel, in the region that surrounds the Gaza Strip, an area which is under ongoing exposure to missile attacks. The sample was stratified by age group (50–64, 65–90), gender, and place of residence (see Palgi, 2016, for further information). The first interview (T1) was conducted with 339 individuals between January and February 2014; however, the current study focused on two subsequent interview waves (T2 and T3) that included assessments of physical functioning.

T2 interviews started on December 2014, 4 months after the 2014 Israel–Gaza conflict (a.k.a. Operation Protective Edge), and lasted until July 2015. During the conflict, more than 4,500 rockets were fired on civilian regions in Israel, especially in its southern region. The sample included 170 participants who completed the interview (71% of those who were interviewed at T1 and who gave their consent to be interviewed again).

T3 interviews were conducted between December 2015 and April 2016. During the period extending from the end of T2 to the end of T3, 22 rockets were fired, and that period was relatively one of the quietest since 2006. The sample included 132 participants who completed the questionnaire at all three waves (thus, these participants had two assessments of the main study variables – PTSD symptoms and self-rated physical functioning – which were examined at T2 and T3). Fourteen of T2 respondents requested not to participate in subsequent interviews. Out of the remaining 156 respondents, 15 declined to be interviewed when approached to during T3, and nine more were not located. Therefore, 84.6% of those interviewed at T2 and who gave consent to be interviewed again participated in T3.

Attrition analyses did not find significant differences in age, gender, education, level of exposure, physical functioning, and subjective age between those who participated at both waves (T2 and T3) compared to those who participated at only the T2 wave. There was a marginally significant difference in T2 PTSD symptoms (*t*[164] = 1.97, *p* = 0.05), with those participating at both times reporting lower symptom levels (*M* = 32.64, *SD* = 16.93) compared to those who only participated at T2 (*M* = 39.16, *SD* = 19.48).

Demographic characteristics of the final sample (*N* = 132) are presented in **Table 1**. The mean age at T2 was 66.90 (*SD* = 9.14, range = 51–88), more than half were women (54.5%) and the mean years of education was 13.52 (*SD* = 3.18).

**Table 1:** Descriptive statistics and correlations between the study variables.

Variable	*M*/%	*SD*	1	2	3	4	5	6	7
1. PTSD T2	32.64	16.93	–						
2. PTSD T3	31.51	16.38	0.70^∗∗∗^	–					
3. Self-rated physical functioning T2	61.85	30.86	-0.48^∗∗∗^	-0.31^∗∗∗^	–				
4. Self-rated physical functioning T3	64.61	33.91	-0.49^∗∗∗^	-0.37^∗∗∗^	0.76^∗∗∗^	–			
5. Subjective age T2	0.16	0.19	-0.30^∗∗^	-0.16	0.34^∗∗∗^	0.30^∗∗^	–		
6. Age T2	66.90	9.14	-0.08	-0.17^∗^	-0.15	-0.26^∗∗^	0.03	–	
7. Gender (women)	54.5	-	0.11	0.09	-0.10	-0.10	-0.07	0.04	–
8. Education	13.52	3.18	-0.23^∗∗^	-0.29^∗∗^	0.22^∗^	0.23^∗∗^	0.13	-0.08	0.01

*N = 132, ^∗^*p* < 0.05, ^∗∗^*p* < 0.01, ^∗∗∗^*p* < 0.001*.

The telephone interviews were carried out by experienced interviewers in either Hebrew or Russian, lasting an average of about 20–30 min. in each wave. Informed consent was obtained at the beginning of each interview. Recruitment and administration for both waves were approved by the Ethics Committee at the University of Haifa.

### Measures

Posttraumatic stress symptoms were assessed in both T2 and T3 using the PTSD Checklist (PCL, Weathers et al., 2013), adapted to the DSM-5 (American Psychiatric Association, 2013). This questionnaire is a 20-item measure of posttraumatic-stress symptoms. For each symptom, participants are asked to choose their response on a 5-point Likert scale from 1 (not at all bothered) to 5 (extremely bothered). Participants were asked to rate each symptom while thinking of the most stressful event related to rocket fire that they reported having been exposed to. Due to the chronic nature of the stressor, the questions referred specifically to symptoms experienced during the previous month. Reliability was excellent (Cronbach’s α = 0.95 in both T2 and T3).

Self-rated physical functioning was assessed at T2 and T3 by the six items assessing the physical health domain from the 12-item Short-Form Health Survey (SF-12; Ware et al., 1996; i.e., general rating of health, having trouble in moderate activities, having trouble in climbing flights of stairs, accomplishing less because of physical health, experiencing limits in work or other activities due to physical health, and experiencing pain that interferes with both housework and work outside home). Scores were transformed according to a formula converting raw scale scores to values representing the percentage of the total possible score achieved, ranging from 0 to 100 (Ware et al., 2002). Higher scores indicate better physical functioning (Cronbach’s α = 0.87 and 0.90 in T2 and T3, respectively).

Subjective age was assessed in T2 by asking participants to state how old they felt most of the time. Subjective age score reflects proportional discrepancy from chronological age – the difference between chronological age and felt age, divided by chronological age (Stephan et al., 2015a). Higher scores reflect a younger age identity.

Covariates included the following demographics: T2 age, gender, and years of education.

### Data Analysis

We used structural equation modeling with AMOS 23, constructing a cross-lagged autoregressive design. The model simultaneously tested a regression path from T2 PTSD to T3 self-rated physical functioning, and from T2 self-rated physical functioning to T3 PTSD, allowing the error terms of the same wave variables to covary. To test whether the reciprocal effects of PTSD and self-rated physical functioning are moderated by subjective age, we included the effect of an interaction term between T2 predictors and subjective age on T3 outcomes in case the effect of T2 predictors was significant. The T2 variables were regressed on the three control variables, which were related to each other. After examining the modification indices, we improved model fit by allowing the error terms of T2 age and physical functioning to covary.

Following the recommendations by Hu and Bentler (1999) for relatively small samples, model fit was assessed by the chi-square value divided by degrees of freedom (χ^2^/*df*), the comparative fit index (CFI), and the standardized root mean squared residual (SRMR). Scores above 0.95 indicate good fit for CFI, and values below 0.08 indicate good fit for SRMR (Hu and Bentler, 1999).

Across variables with missing values, 0.8–7.6% cases were missing. Little’s missing completely at random test revealed that the data was missing completely at random, χ^2^(25) = 32.05, *p* = 0.15. Missing data were handled with maximum likelihood *via* the AMOS 23.

## Results

**Table 1** presents the descriptive statistics for the study variables. As can be seen, PTSD correlated strongly across times, *r* = 0.70, *p* < 0.001, as did self-rated physical functioning, *r* = 0.76, *p* < 0.001. PTSD and self-rated physical functioning negatively correlated at both times (for T2: *r* = -0.48, *p* < 0.001; for T3: *r* = -0.37, *p* < 0.001). Moreover, the two variables moderately correlated across times (T2 PTSD-T3 self-rated physical functioning: *r* = -0.49, *p* < 0.001; T2 self-rated physical functioning-T3 PTSD: *r* = -0.37, *p* < 0.001). Subjective age at T2 significantly correlated with T2 PTSD (*r* = -0.30, *p* < 0.01) and with self-rated physical functioning at both times (*r* = 0.30–0.34, *p* < 0.01). Finally, covariates showed rather small correlations with the main variables (absolute value *r* ranged 0.03 to 0.29).

When first performing the cross-lagged model without subjective age and its potential interaction with T2 predictors, the model exhibited good model fit, χ^2^/*df* = 2.69, CFI = 0.96, SRMR = 0.04. The coefficients showed that T2 PTSD predicted T3 self-rated physical functioning (*B* = -0.52, *se* = 0.13, β = -0.26, *p* = 0.03), but T2 self-rated physical functioning did not significantly predict T3 PTSD (*B* = -0.002, *se* = 0.04, β = -0.003, *p* = 0.96).

**FIGURE 1:**
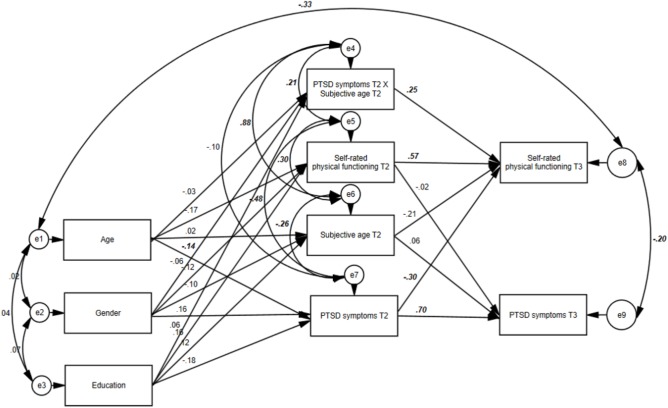
The final model including the reciprocal effects of PTSD symptoms and self-rated health and the main effect of subjective age, and its interaction with T2 PTSD symptoms (values represent standardized coefficients; values in bold italics refer to significant coefficients, *p* < 0.05).

We next added subjective age and its interaction with T2 PTSD on T3 self-rated physical functioning to the model. The model exhibited good fit, χ^2^/*df* = 3.04, CFI = 0.97, SRMR = 0.03. **Table 2** presents the selected parameters for that model. **Figure 1** presents all parameters for that model. Again, T2 PTSD predicted T3 self-rated physical functioning, but not *vice versa*. Moreover, there was a significant interaction between T2 PTSD and T2 subjective age predicting T3 self-rated physical functioning.^1^

**Table 2:** Selected parameters for the cross-lagged models.

			Main model (all respondents)			Younger subjective age			Older subjective age		
Covariance			B	SE	β	B	SE	β	B	SE	β
PTSD T2	↔	Self-rated physical functioning T2	-0.48**	0.08	-	-0.34*	0.11	-	-0.55*	0.10	-
Self-rated physical functioning T3	↔	PTSD T3	-0.20	0.10	-	-0.21	0.14	-	-0.18	0.15	-
**Regression weights**											
PTSD T2	→	PTSD T3	0.70**	0.07	0.70	0.71**	0.07	0.62	0.71**	0.10	0.78
Self-rated physical functioning T2	→	Self-rated physical functioning T3	0.63**	0.07	0.57	0.61*	0.07	0.58	0.61*	0.09	0.55
PTSD T2	→	Self-rated physical functioning T3	**-0.59^∗^**	**0.14**	**-0.30**	**-0.32**	**0.18**	-**0.16**	**-0.63^∗∗^**	**0.19**	-**0.33**
Self-rated physical functioning T2	→	PTSD T3	**-0.01**	**0.04**	**-0.02**	-**0.09**	**0.05**	**-0.15**	**0.08**	**0.05**	**0.15**
Subjective age T2	→	PTSD T3	5.00	6.21	0.06						
Subjective age T2	→	Self-rated physical functioning T3	-36.39	22.56	-0.21						
PTSD T2^∗^subjective age T2	→	Self-rated physical functioning T3	1.27*	0.64	0.25						

*The coefficients for background characteristics are not presented in the table, but are available in **Figure 1**. Coefficients in bold represent cross-lagged effects. ^∗^*p* < 0.05, ^∗∗^*p* < 0.01, ^∗∗∗^*p* < 0.001*.

**FIGURE 2:**
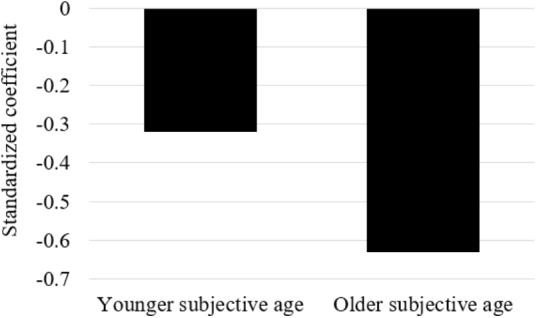
The effect of T2 PTSD on T3 self-rated physical functioning (standardized coefficient) in both subjective age groups.

In order to understand the nature of the interaction, we divided our samples into two groups according to the median subjective age score (*Md* = 0.12) to those who felt relatively younger and those who felt relatively older. We next assessed the reciprocal effects of PTSD and self-rated physical functioning in each group (see **Table 2**). The effect of T2 PTSD on T3 self-rated physical functioning was *B* = -0.32, β = -0.16, *p* = 0.08, and *B* = -0.63, β = -0.33, *p* < 0.01, for those with relatively younger subjective age and those with relatively older subjective age, respectively. **Figure 2** presents the effect of T2 PTSD on T3 self-rated physical functioning in both subjective age groups.

We then constrained the path between T2 PTSD and T3 self-rated physical functioning to be equal between the groups, and compared the fit indices between the constrained and unconstrained models. Results suggested that for the T2 PTSD-T3 self-rated physical functioning path, the unconstrained model (χ^2^/*df* = 1.50, CFI = 0.97, SRMR = 0.04) fitted the data significantly better than the constrained model (χ^2^/*df* = 1.83, CFI = 0.94, SRMR = 0.06), Δχ^2^ = 7.58, *p* = 0.02. The latter results again indicate that the PTSD-self-rated physical symptoms relationship is not the same for participants who felt relatively older and younger. Namely, as above, it is stronger for those feeling older.

## Discussion

Partially supporting our hypotheses, we found among Israeli middle-aged and older adults exposed to ongoing missile attacks that higher PTSD symptoms predicted lower self-rated physical functioning approximately 1 year later. The reverse effect was not significant, meaning that self-rated physical functioning at baseline did not predict subsequent PTSD symptoms. Moreover, subjective age moderated the effect of T2 PTSD symptoms on T3 self-rated physical functioning. PTSD symptoms had a greater effect on self-rated physical functioning among individuals who felt relatively older compared to those who felt relatively younger. Actually, the effect of PTSD symptoms on self-rated physical functioning was non-significant among those who felt 12% younger than their age or younger than that. We now turn to discuss the findings in more detail.

The current study helps clarify the temporal sequencing of PTSD and physical functioning, as it joins previous studies that mainly supported the effect of PTSD symptoms on later physical health, but less so regarding the reverse effect, whereby physical functioning contributes to the development or aggravation of PTSD symptoms. The current results are in line with previous findings showing that PTSD predicted a higher chance of developing type 2 diabetes (Roberts et al., 2015), higher physical disability (Schweininger et al., 2015), greater physical pain (Valentine et al., 2016), and lower physical functioning (Ramchand et al., 2008). The predictive utility of physical health after traumatic exposure on later PTSD symptoms was less frequently supported (Ramchand et al., 2008). It seems that a rather consistent pattern of temporal sequencing emerged across various traumatic events, in different populations, across short- and long-time spans and with several physical health indices. However, as most studies focused on young adults and used self-report measures, there is still a need to assess the reciprocal effects of PTSD symptoms and physical health among older adults by using objective indices.

The current study further looked at variables that may moderate the reciprocal effects between PTSD symptoms and self-rated physical functioning, focusing on subjective age. Older subjective age aggravated the effect of PTSD symptoms on self-rated physical functioning. The current finding joins others in pointing to the potential of subjective age to mitigate or increase effects of other variables. Accordingly, older subjective age was previously shown to increase the association between PTSD symptoms and successful aging (Shrira et al., 2016), the association of feeling close to death with psychological distress (Shrira et al., 2014), and the association between depressive symptoms and cognitive impairment (Choi et al., 2017). The current finding mostly complemented those presented by Segel-Karpas et al. (2017), where depressive symptoms showed a much stronger effect on subsequent medical conditions among those with an older age identity at baseline.

The findings can be further understood in light of the recovery capital framework (Cloud and Granfield, 2008). According to White and Cloud (2008), human capital includes personal values and beliefs, aspirations and hope, and other personal resources that will enable the individual to prosper. Subjective age is a personal belief relating to one’s age identity and therefore may be regarded as an element of human capital. Therefore, a young subjective age may be considered as recovery capital largely reflecting a perception that one has sufficient resources to deal with the traumatic event and its repercussions (Hoffman et al., 2016). A relatively old age identity may reflect a perception that one is not equipped to cope with trauma and its aftermath. Thus, an older subjective age can associate with other negative perceptions that are likely to appear in PTSD, such as perceiving oneself as powerless, inferior, or futureless (Brewin, 2003), or perceiving the trauma as highly central to one’s identity and life story (Gehrt et al., 2018). The potential associations between an older subjective age and other negative perceptions should be assessed in future studies in order to better understand whether age identity has a unique effect or can be conceptualized as part of the abovementioned negative perceptions.

The perception of oneself as old might moderate the PTSD-physical health association and increase the effect of PTSD symptoms on physical functioning due to multiple reasons. An older subjective age may relate to psychological, biological, and behavioral factors that may amplify the detrimental effect of PTSD symptoms, and indeed previous studies showed that an old age identity is a unique concomitant of depression, anxiety (Shrira et al., 2014), indices of biological vulnerability (Lahav et al., 2018; Stephan et al., 2015a,b; Stephan et al., 2017), and health risk behaviors (Wienert et al., 2017). Moreover, an older subjective age can suggest the internalization of age stereotypes and viewing psychopathology and physical morbidity as an unavoidable part of late-life decline that one must succumb to.

It is possible that subjective age moderated the effect of PTSD on self-rated physical health, as it merely reflected health problems that would potentially get worse under PTSD. Yet, numerous works have shown that older age identity predicts prospective adverse health outcomes even after controlling for baseline health measures (see reviews, Kotter-Grühn et al., 2015; Wurm et al., 2017). Accordingly, a meta-analysis study found that feeling older predicted higher chances of a future medical event, future hospitalization, and higher mortality rates after controlling for demographics, psychosocial functioning, and baseline health-related variables (Westerhof et al., 2014). Therefore, it is reasonable to assume that subjective age does not simply mirror health deterioration due to stress, but rather has its own unique and additive effect.

Before moving to practical implications, it should be emphasized that relatively few studies found a moderation effect for subjective age (Choi et al., 2017; Segel-Karpas et al., 2017; Shrira et al., 2014, 2016), and this is the first study showing subjective age to moderate the longitudinal effect of PTSD symptoms on self-rated physical health. Therefore, future studies should include *a priori* hypothesis with regard to moderation effect of subjective age and replicate our findings before drawing firm conclusions. Nevertheless, based on the current state of evidence, our results point to the need to develop interventions aimed at coping with an older age identity and facilitating a younger age identity among traumatized older individuals. This may strengthen human capital which in turn may have a positive effect also on other types of recovery capital. It may also be beneficial to monitor psychological, biological, behavioral, and attentional factors that can link an older age identity with subsequent physical decline. For example, one can closely track the health behaviors of posttraumatic older adults with older subjective age, as they might be prone to neglecting health-promoting behavior and self-care, thus exacerbating the negative effect of PTSD on their physical health (cf. Wienert et al., 2017). Moreover, it may be speculated that such interventions can tackle more general negative perceptions and stereotypes these individuals may possess, so as to increase their sense of self-efficacy and their motivation to address their illnesses.

The findings of this study should be assessed in light of its limitations. A main limitation of this study is that our indicators of PTSD symptoms and physical functioning were based on self-report. As noted, most previous studies that assess the reciprocal effects of PTSD and physical health relied on self-report measures (but see Roberts et al., 2015 for an exception), and there is a special need to look at more objective measures such as the psychiatric diagnosis of PTSD and physician-diagnosed medical conditions or biological markers, or examine older adults who have experienced explicit health events. Still, our measure of physical functioning was taken from a short form of the SF-36, which is considered the most widely used generic health outcome instrument throughout the world (Turner-Bowker and Hogue, 2014). The SF-12 retains strong psychometric performance and was found to be most useful in measuring and monitoring health outcomes for both general and disease-specific populations (Turner-Bowker and Hogue, 2014; Ware et al., 1996). Moreover, subjective age was assessed using a single item. Although this is the most frequent method to assess this variable (see reviews in Diehl et al., 2014; Kotter-Grühn et al., 2015; Wurm et al., 2017), recent works build on the seminal work of Kastenbaum et al. (1972), and assess subjective age in regard to multiple domains, asking individuals to which age group would they compare themselves within the domain of family life, social relations, leisure, personality, physical health, mental fitness, appearance, etc. (e.g., Kornadt et al., 2016). Finally, despite its advantages, the longitudinal design does not allow certainty in interpretation of causality. Other variables, such as personality traits (e.g., neuroticism) or genetic predisposition, could increase one’s vulnerability to both PTSD symptoms and physical morbidity, thereby resulting in older subjective age.

Despite these limitations, this study contributes to the literature. By using a longitudinal design and examining the role of subjective age in the relationships between PTSD symptoms and self-rated physical functioning, we highlight a possible factor that shapes these relationships, weakening the association for some older adults, and strengthening it for others.

## Author Contributions

All authors took part in planning the theoretical and conceptual basis for the study. AS performed the statistical analyses and wrote the first draft of the paper. All authors took part in critically reviewing and editing the manuscript.

## Conflict of Interest Statement

The authors declare that the research was conducted in the absence of any commercial or financial relationships that could be construed as a potential conflict of interest.

## Footnotes

1 When running the model without controlling for any background characteristics, the results remained similar. The coefficients showed that T2 PTSD predicted T3 self-rated physical functioning (*B* = -0.48, *se* = 0.14, *p* < 0.001), but T2 self-rated physical functioning did not significantly predict T3 PTSD (*B* = -0.009, *se* = 0.04, *p* = 0.83). Moreover, there was a significant interaction between T2 PTSD and T2 subjective age predicting T3 self-rated physical functioning (*B* = 1.36, *se* = 0.67, *p* = 0.04).

## References

[B1] American Psychiatric Association (2013). *Diagnostic and Statistical Manual of Mental Disorders (DSM-5^®^)*. Washington, DC American Psychiatric Pub.

[B2] Avidor S. Benyamini Y. Solomon Z. (2016). Subjective age and health in later life: the role of posttraumatic symptoms. *J. Gerontol. B Psychol. Sci. Soc. Sci.* 71 415–424. 10.1093/geronb/gbu150 25324296

[B3] Barak B. (1987). Cognitive age: a new multidimensional approach to measuring age identity. *Int. J. Aging Hum. Dev.* 25 109–128. 10.2190/RR3M-VQT0-B9LL-GQDM 3436682

[B4] Barak B. (2009). Age identity: a cross-cultural global approach. *Int. J. Behav. Dev.* 33 2-11. 10.1177/0165025408099485

[B5] Brewin C. R. (2003). *Posttraumatic Stress Disorder: Malady or Myth?* New Haven, CT: Yale University Press.

[B6] Choi E. Y. Kim Y. S. Lee H. Y. Shin H. R. Park S. Cho S. E. (2017). The moderating effect of subjective age on the association between depressive symptoms and cognitive functioning in Korean older adults. *Aging Ment. Health* 10.1080/13607863.2017.1390733 [Epub ahead of print]. 29052424

[B7] Chopik W. J. Bremner R. H. Johnson D. J. Giasson H. L. (2018). Age differences in age perceptions and developmental transitions. *Front. Psychol.* 9:67. 10.3389/fpsyg.2018.00067 29449823 PMC5799826

[B8] Cleveland J. N. Shore L. M. Murphy K. R. (1997). Person-and context-oriented perceptual age measures: additional evidence of distinctiveness and usefulness. *J. Organ. Behav.* 18 239–251. 10.1002/(SICI)1099-1379(199705)18:3<239::AID-JOB794>3.0.CO;2-A

[B9] Cloud W. Granfield R. (2008). Conceptualizing recovery capital: expansion of a theoretical construct. *Subst. Use Misuse* 43 1971–1986. 10.1080/10826080802289762 19016174

[B10] Cook J. M. Simiola V. (2017). Trauma and PTSD in older adults: prevalence, course, concomitants and clinical considerations. *Curr. Opin. Psychol.* 14 1–4. 10.1016/j.copsyc.2016.08.003 28813305

[B11] Diehl M. Wahl H. W. Barrett A. E. Brothers A. F. Miche M. Montepare J. M. (2014). Awareness of aging: theoretical considerations on an emerging concept. *Dev. Rev.* 34 93–113. 10.1016/j.dr.2014.01.001 24958998 PMC4064469

[B12] Gehrt T. B. Berntsen D. Hoyle R. H. Rubin D. C. (2018). Psychological and clinical correlates of the centrality of event scale: a systematic review. *Clin. Psychol. Rev.* 65 57–80. 10.1016/j.cpr.2018.07.006 30138786 PMC6291852

[B13] Granfield R. Cloud W. (1999). *Coming Clean: Overcoming Addiction without Treatment*. New York, NY: NYU Press.

[B14] Granfield R. Cloud W. (2001). Social context and “natural recovery”: the role of social capital in the resolution of drug-associated problems. *Subst. Use Misuse* 36 1543–1570. 10.1081/JA-100106963 11693955

[B15] Grieger T. A. Cozza S. J. Ursano R. J. Hoge C. Martinez P. E. Engel C. C. (2006). Posttraumatic stress disorder and depression in battle-injured soldiers. *Am. J. Psychiatry* 163 1777–1783. 10.1176/ajp.2006.163.10.1777 17012689

[B16] Hoffman Y. S. Shrira A. Cohen-Fridel S. Grossman E. S. Bodner E. (2016). Posttraumatic stress disorder symptoms as a function of the interactive effect of subjective age and subjective nearness to death. *Pers. Individ. Differ.* 102 245–251. 10.1016/j.paid.2016.07.017

[B17] Hu L. Bentler P. M. (1999). Cutoff criteria for fit indexes in covariance structure analysis: conventional criteria versus new alternatives. *Struct. Equ. Model.* 6 1–55. 10.1080/10705519909540118

[B18] Kastenbaum R. Derbin V. Sabatini P. Artt S. (1972). “The ages of me”: toward personal and interpersonal definitions of functional aging. *Int. J. Aging Hum. Dev.* 3 197–211. 10.2190/TUJR-WTXK-866Q-8QU7

[B19] Kornadt A. E. Hess T. M. Voss P. Rothermund K. (2016). Subjective age across the life span: a differentiated, longitudinal approach. *J. Gerontol. B Psychol. Sci. Soc. Sci.* 73 767–777 10.1093/geronb/gbw072 27334638

[B20] Kotter-Grühn D. Hess T. M. (2012). The impact of age stereotypes on self-perceptions of aging across the adult lifespan. *J. Gerontol. B Psychol. Sci. Soc. Sci.* 67 563–571. 10.1093/geronb/gbr153 22367710 PMC3441190

[B21] Kotter-Grühn D. Kornadt A. E. Stephan Y. (2015). Looking beyond chronological age: current knowledge and future directions in the study of subjective age. *Gerontology* 62 86–93. 10.1159/000438671 26302678

[B22] Lahav Y. Avidor S. Stein J. Y. Zhou X. Solomon Z. (2018). Telomere length and depression among ex-prisoners of war: the role of subjective age. *J. Gerontol. B Psychol. Sci. Soc. Sci.* 10.1093/geronb/gby006 [Epub ahead of print]. 29415270

[B23] Lee S. Y. Park C. L. (2018). Trauma exposure, posttraumatic stress, and preventive health behaviours: a systematic review. *Health Psychol. Rev.* 12 75–109. 10.1080/17437199.2017.1373030 28854859

[B24] Levy B. (2009). Stereotype embodiment: a psychosocial approach to aging. *Curr. Dir. Psychol. Sci.* 18 332–336. 10.1111/j.1467-8721.2009.01662.x 20802838 PMC2927354

[B25] Lohr J. B. Palmer B. W. Eidt C. A. Aailaboyina S. Mausbach B. T. Wolkowitz O. M. (2015). Is post-traumatic stress disorder associated with premature senescence? A review of the literature. *Am. J. Geriatr. Psychiatry* 23 709–725. 10.1016/j.jagp.2015.04.001 25959921 PMC4568841

[B26] Pacella M. L. Hruska B. Delahanty D. L. (2013). The physical health consequences of PTSD and PTSD symptoms: a meta-analytic review. *J. Anxiety Disord.* 27 33–46. 10.1016/j.janxdis.2012.08.004 23247200

[B27] Palgi Y. (2016). Subjective age and distance-to-death moderate the association between posttraumatic stress symptoms and posttraumatic growth among older adults. *Aging Ment. Health* 20 948–954. 10.1080/13607863.2015.1047320 26028224

[B28] Ramchand R. Marshall G. N. Schell T. L. Jaycox L. H. (2008). Posttraumatic distress and physical functioning: a longitudinal study of injured survivors of community violence. *J. Consult. Clin. Psychol.* 76 668–676. 10.1037/0022-006X.76.4.668 18665694 PMC3678762

[B29] Rheingold A. A. Acierno R. Resnick H. S. (2004). “Trauma, posttraumatic stress disorder, and health risk behaviors,” in *Trauma and Health: Physical Health Consequences of Exposure to Extreme Stress*, eds Schnurr P. P. Green B. L. (Washington, DC: American Psychological Association), 217–243. 10.1037/10723-009

[B30] Roberts A. L. Agnew-Blais J. C. Spiegelman D. Kubzansky L. D. Mason S. M. Galea S. (2015). Posttraumatic stress disorder and incidence of type 2 diabetes mellitus in a sample of women: a 22-year longitudinal study. *JAMA Psychiatry* 72 203–210. 10.1001/jamapsychiatry.2014.2632 25565410 PMC4522929

[B31] Schnurr P. P. (2017). “Physical health and health services utilization,” in *APA Handbook of Trauma Psychology*, eds Gold S. Cook J. Dahlenberg D. (Washington, DC: American Psychological Association), 349–370.

[B32] Schnurr P. P. Green B. L. (2004). “Understanding relationships among trauma, posttraumatic stress disorder, and health outcomes,” in *Trauma and Health: Physical Health Consequences of Exposure to Extreme Stress*, eds Schnurr P. P. Green B. L. (Washington, DC: American Psychological Association), 247—275. 10.1037/10723-010

[B33] Schweininger S. Forbes D. Creamer M. McFarlane A. C. Silove D. Bryant R. A. (2015). The temporal relationship between mental health and disability after injury. *Depress. Anxiety* 32 64–71. 10.1002/da.22288 24995589

[B34] Segel-Karpas D. Palgi Y. Shrira A. (2017). The reciprocal relationship between depression and physical morbidity: the role of subjective age. *Health Psychol.* 36 848–851. 10.1037/hea0000542 28737414 PMC5666207

[B35] Shrira A. Bodner E. Palgi Y. (2014). The interactive effect of subjective age and subjective distance-to-death on psychological distress of older adults. *Aging Ment. Health* 18 1066–1070. 10.1080/13607863.2014.915925 24831662

[B36] Shrira A. Palgi Y. Ben-Ezra M. Hoffman Y. Bodner E. (2016). A youthful age identity mitigates the effect of post-traumatic stress disorder symptoms on successful aging. *Am. J. Geriatr. Psychiatry* 24 174–175. 10.1016/j.jagp.2015.07.006 26560506

[B37] Solomon Z. Greene T. Ein-Dor T. Zerach G. Benyamini Y. Ohry A. (2014). The long-term implications of war captivity for mortality and health. *J. Behav. Med.* 37 849–859. 10.1007/s10865-013-9544-3 24165831

[B38] Solomon Z. Helvitz H. Zerach G. (2009). Subjective age, PTSD and physical health among war veterans. *Aging Ment. Health* 13 405–413. 10.1080/13607860802459856 19484604

[B39] Stephan Y. Caudroit J. Chalabaev A. (2011). Subjective health and memory self-efficacy as mediators in the relation between subjective age and life satisfaction among older adults. *Aging Ment. Health* 15 428–436. 10.1080/13607863.2010.536138 21500009

[B40] Stephan Y. Sutin A. R. Terracciano A. (2015a). How old do you feel? The role of age discrimination and biological aging in subjective age. *PLoS One* 10:e0119293. 10.1371/journal.pone.0119293 25738579 PMC4349738

[B41] Stephan Y. Sutin A. R. Terracciano A. (2015b). Younger subjective age is associated with lower C-reactive protein among older adults. *Brain Behav. Immun.* 43, 33–36. 10.1016/j.bbi.2014.07.019 *PMID:25108213* 25108213

[B42] Stephan Y. Sutin A. R. Terracciano A. (2017). Subjective age and cystatin C among older adults. *J. Gerontol. B Psychol. Sci. Soc. Sci.* 10.1093/geronb/gbx124 [Epub ahead of print]. 29045722 PMC6377033

[B43] Turner-Bowker D. Hogue S. J. (2014). “Short form 12 health survey (SF-12),” in *Encyclopedia of Quality of Life and Well-Being Research*, ed. Michalos A. C. (Dordrecht: Springer), 5954–5957. 10.1007/978-94-007-0753-5_2698

[B44] Valentine S. E. Gerber M. W. Nobles C. J. Shtasel D. L. Marques L. (2016). Longitudinal study of mental health and pain-related functioning following a motor vehicle collision. *Health Psychol.* 35 742-750. 10.1037/hea0000329 26998734 PMC5031508

[B45] Ware J. E. Kosinski M. Keller S. D. (1996). A 12-item short-form health survey: construction of scales and preliminary tests of reliability and validity. *Med. Care* 34 220–233. 10.1097/00005650-199603000-00003 8628042

[B46] Ware J. E. Kosinski M. Turner-Bowker D. M. Gandek B. (2002). *User’s Manual for the SF-12v2^®^ Health Survey with a Supplement Documenting SF-12^®^ Health Survey.* Lincoln, RI: Quality Metric Incorporated.

[B47] Weathers F. W. Litz B. T. Keane T. M. Palmieri P. A. Marx B. P. Schnurr P. P. (2013). *The PTSD Checklist for DSM-5 (PCL-5). Scale Available from the National Center for PTSD.* Available at www.ptsd.va.gov

[B50] Westerhof G. J. Miche M. Brothers A. F. Barrett A. E. Diehl M. Montepare J. M. (2014). The influence of subjective aging on health and longevity: a meta-analysis of longitudinal data. *Psychol. Aging* 29 793–802. 10.1037/a0038016 25365689

[B48] Westerhof G. J. Whitbourne S. K. Freeman G. P. (2012). The aging self in a cultural context: the relation of conceptions of aging to identity processes and self-esteem in the United States and the Netherlands. *J. Gerontol. B Psychol. Sci. Soc. Sci.* 67 52–60. 10.1093/geronb/gbr075 21785004

[B49] White W. Cloud W. (2008). Recovery capital: a primer for addiction professionals. *Counselor* 9 22–27.

[B51] Wienert J. Gellert P. Lippke S. (2017). Physical activity across the life-span: does feeling physically younger help you to plan physical activities? *J. Health Psychol.* 22 324–335. 10.1177/1359105315603469 26430064

[B52] Williamson J. B. Porges E. C. Lamb D. G. Porges S. W. (2015). Maladaptive autonomic regulation in PTSD accelerates physiological aging. *Front. Psychol.* 5:1571. 10.3389/fpsyg.2014.01571 25653631 PMC4300857

[B53] Wurm S. Diehl M. Kornadt A. E. Westerhof G. J. Wahl H. W. (2017). How do views on aging affect health outcomes in adulthood and late life? Explanations for an established connection. *Dev. Rev.* 46 27–43. 10.1016/j.dr.2017.08.002 PMC8081396 33927468

[B54] Yehuda R. Hoge C. W. McFarlane A. C. Vermetten E. Lanius R. A. Nievergelt C. M. (2015). Post-traumatic stress disorder. *Nat. Rev. Dis. Primers* 1 1–22. 10.1038/nrdp.2015.57 27189040

